# Comparison of library preparation protocols and bioinformatic pipelines in high-throughput 16S rRNA gene sequencing

**DOI:** 10.1186/s12866-026-05344-6

**Published:** 2026-07-01

**Authors:** Olivia Andersson, Anna Fagerström, Katharina Dannenberg, Jan Kekki, Julia Rode, Ignacio Rangel, Carl Mårten Lindqvist, Bianca Stenmark

**Affiliations:** 1 Clinical Research Center, Faculty of Medicine and Health, Örebro University, Örebro, SE- 701 85 Sweden; 2 Clinical Genomics Örebro, Science for Life Laboratory, Örebro University, Örebro, SE-701 85 Sweden; 3 School of Medical Sciences, Faculty of Medicine and Health, Örebro University, Örebro, SE-701 85 Sweden; 4 Food and Health Centre, Örebro University, Örebro, SE-701 85 Sweden

**Keywords:** 16S rRNA sequencing, Microbial profiling, Bioinformatics, Library preparation, Amplicon region, Taxonomic resolution

## Abstract

**Background:**

16S rRNA gene sequencing is widely used for bacterial community profiling in both clinical and research contexts. The expanding availability of library preparation protocols and bioinformatic pipelines increases analytical flexibility but may also introduce method-dependent biases that affect inferred microbial composition and relative abundance estimates. The relative impact of library preparation protocol, amplicon region, and bioinformatic pipeline on species-level taxonomic inference and compositional agreement remains insufficiently characterised. We therefore compared the Illumina 16S Metagenomic Sequencing Library Preparation protocol (V3–V4) and the Zymo Quick-16S Plus NGS Library Prep Kit (V1–V2 and V3–V4) in combination with two bioinformatic pipelines, nf-core/ampliseq and TRANA. Performance was assessed using defined microbial community standards and human faecal and colonic biopsy samples.

**Results:**

Pipeline choice was the dominant driver of variation in inferred community composition, exceeding the effects of amplicon regions and library preparation protocols. Genus-level profiles were broadly concordant across methods. Species-level resolution and agreement with expected community composition differed systematically between pipelines, with TRANA demonstrating lower Bray–Curtis dissimilarities to expected compositions than nf-core/ampliseq. Amplicon region had a secondary, pipeline-dependent effect, while protocol differences were minor. In clinical samples, inter-individual biological variation exceeded technical variation.

**Conclusions:**

Bioinformatic processing substantially influenced species-level inference in short-read 16S sequencing, highlighting the importance of pipeline selection for microbiome study design and cross-study comparability.

**Supplementary Information:**

The online version contains supplementary material available at 10.1186/s12866-026-05344-6.

## Background

The characterisation of microbial communities in complex biological samples is facilitated by high-throughput sequencing approaches. In clinical microbiome research, microbial profiling provides insights into community composition that support pathogen detection and the study of microbiota-associated conditions, including gastrointestinal disorders [1, 2]. Two sequencing strategies are widely used: targeted amplicon sequencing and shotgun metagenome sequencing. Shotgun metagenomics non-selectively sequences genomic material within a sample, enabling simultaneous taxonomic and functional profiling. Its feasibility in clinical contexts is limited by high proportions of host DNA contamination [3], often necessitating host-depletion methods that may inadvertently alter microbial profiles [4] and deeper sequencing to achieve adequate coverage, particularly in low-biomass samples [3, 5]. Consequently, targeted amplicon sequencing remains widely adopted due to lower cost, reduced susceptibility to host DNA contamination, lower computational demands, and availability of well-established reference databases [6, 7].

Targeted amplicon sequencing selectively amplifies and sequences taxonomic marker genes, most frequently the 16S ribosomal RNA (16S rRNA) gene. Present in all bacteria and archaea, the 16S rRNA gene is a highly conserved marker containing nine hypervariable regions (V1-V9), which provide discriminatory power for taxonomic classification. 16S rRNA sequencing typically targets one or more of these regions, with the choice of region having a significant impact on taxonomic resolution by capturing varying levels of phylogenetic diversity [6–9].

The selection of hypervariable region is one of several methodological variables that shape microbial profiling outcomes in 16S rRNA sequencing. Workflow choices, including library preparation protocol and bioinformatic pipeline, can impact taxonomic resolution, detection sensitivity, and relative abundance estimates. Differences in primers, reference databases, and processing and filtering steps can alter microbial signatures and hinder comparability across studies and efforts toward workflow standardisation [2, 6, 7]. Commercially available library preparation protocols such as the 16S Metagenomic Sequencing Library Preparation protocol (Illumina) [10], which targets the V3–V4 region, are widely adopted but involve labour-intensive steps. Alternative kits such as the Quick-16S Plus NGS Library Prep Kit (Zymo Research) offer a more streamlined workflow, with reduced hands-on time and support for targeting both the V1–V2 and V3–V4 regions [11].

For processing, multiple bioinformatic pipelines are available, employing varied strategies for taxonomic classification. Two publicly available pipelines, nf-core/ampliseq (https://nf-co.re/ampliseq/) and TRANA (previously known as GMS_16S, https://github.com/genomic-medicine-sweden/TRANA), exemplify different analytical approaches. nf-core/ampliseq supports multiple classifiers and reference databases, with taxonomic assignment by default performed using DADA2 in combination with the SILVA database [12]. In contrast, TRANA, developed within the Genomic Medicine Sweden (GMS) initiative, uses EMU for taxonomic classification, with its bundled reference database comprising sequences from NCBI 16S RefSeq and the Ribosomal RNA Operon Copy Number database (rrnDB) [13, 14].

Despite the widespread use of 16S rRNA sequencing, systematic evaluations of how methodological choices, specifically the selection of library preparation protocol, targeted 16S rRNA hypervariable region, and bioinformatic pipeline, influence taxonomic resolution remain limited. Independent assessments in clinically derived samples are particularly scarce, and evaluation of bioinformatic performance on short-read 16S rRNA sequencing is essential for informed pipeline selection. To address these gaps, we performed a systematic comparison of protocols, amplicon regions, and pipelines using both defined microbial standards and clinical samples, enabling evaluation of their impacts on downstream microbial profiling.

## Materials and methods

### Study design

**Fig. 1:**
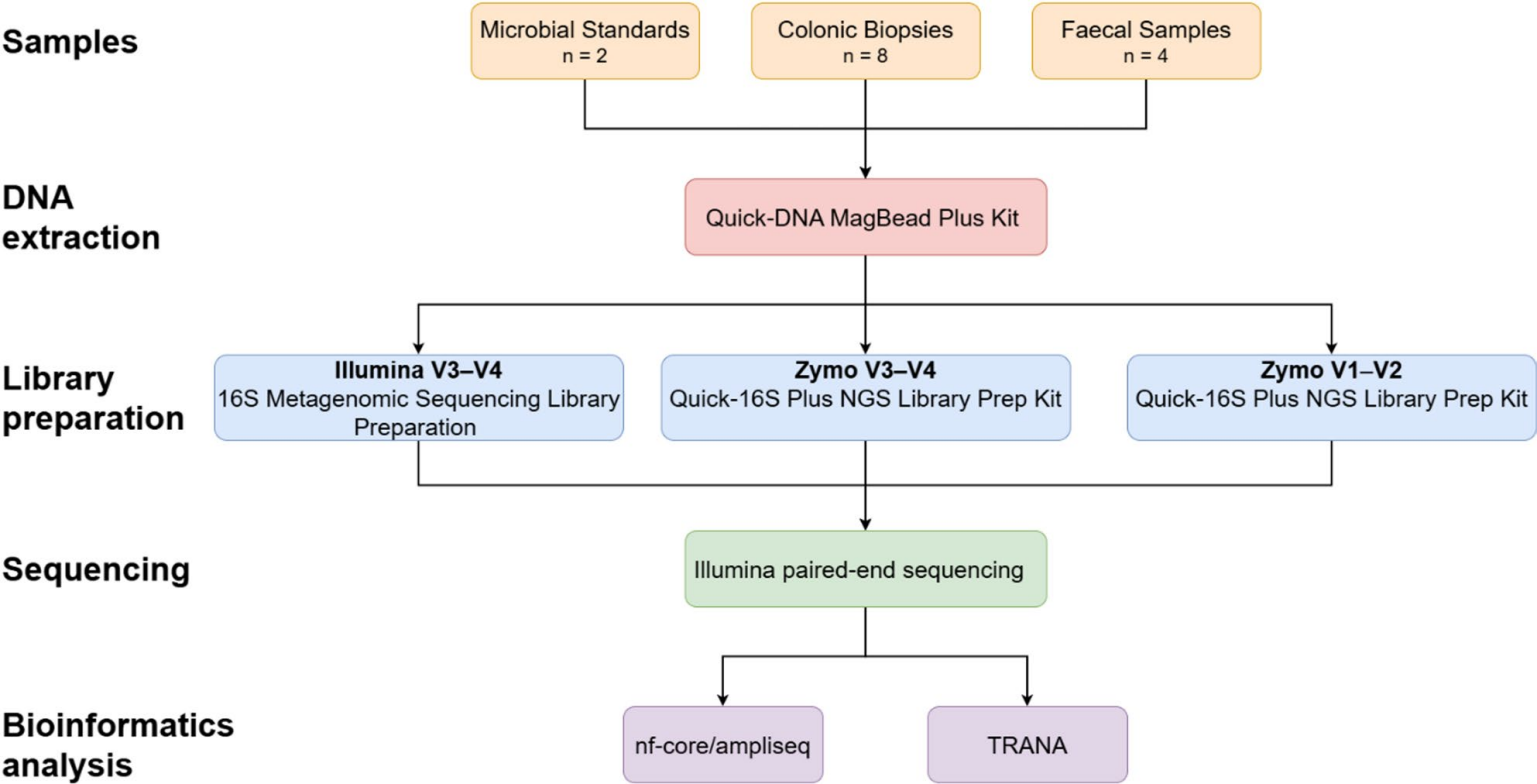
Schematic overview of the study design. Microbial standards (ZymoBIOMICS Gut Microbiome Standard (D6331) and ZymoBIOMICS Microbial Community Standard (D6300)), colonic biopsies, and faecal samples were extracted using the Quick-DNA MagBead Plus Kit. Amplicon libraries were prepared using the 16S Metagenomic Sequencing Library Preparation protocol (Illumina; V3–V4) or the Quick-16S Plus NGS Library Prep Kit (Zymo Research; V1–V2 or V3–V4, unique dual indexes (UDI)). Libraries were sequenced on an Illumina NextSeq platform, and resulting reads were analysed using nf-core/ampliseq and TRANA

This study compared two 16S rRNA gene library preparation workflows using microbial standards and clinical faecal and colonic biopsy samples, selected for their relevance to gut microbiota profiling. The Quick-16S Plus NGS Library Prep Kit (Zymo Research; hereafter referred to as the Zymo Protocol) was evaluated for both the V1–V2 and V3–V4 regions of the 16S rRNA gene, whereas the 16S Metagenomic Sequencing Library Preparation protocol (Illumina; hereafter referred to as the Illumina Protocol) was used exclusively for the V3–V4 region, as the V1–V2 region has not been validated for this protocol. Sequencing reads were processed using two bioinformatic pipelines, TRANA and nf-core/ampliseq. An overview of the study design is provided in Fig. 1.

### Sample collection

This study included microbial standards (ZymoBIOMICS Gut Microbiome Standard (D6331) [15] and ZymoBIOMICS Microbial Community Standard (D6300) [16]; Zymo Research, Irvine, CA, USA), comprising defined microbial agents (Supplementary Table 1), to assess detection sensitivity and taxonomic resolution. D6300 has relatively uniform abundances, whereas D6331 mimics the human gut microbiome and exhibits a staggered abundance distribution. For the V3–V4 region, five replicates of D6331 and four replicates of D6300 were included, whereas four replicates of each standard were included for the V1–V2 region. In addition, anonymised faecal samples (*n* = 4, prepared in duplicate for the V3–V4 region) and colonic biopsies (*n* = 8) from healthy individuals enrolled in a larger cohort study conducted at Örebro University Hospital between April 2019 and March 2022 (fresh-frozen in DNA/RNA Shield) were included to assess methodological feasibility in clinical material with unknown microbial composition, and were selected based on availability. 

### DNA extraction

The Quick-DNA MagBead Plus Kit (Zymo Research) was used for DNA extraction, with purification performed according to manufacturer’s protocol and modifications limited to bead-beating prior to enzymatic lysis, as described below. Matrix-specific lysis conditions were used to account for differences in sample composition and biomass between faecal material, colonic biopsies, and microbial standards. A negative extraction control consisting of DNA/RNA Shield (Zymo Research) was included in each extraction batch.

#### Faecal samples

Were bead-beaten in Lysing Matrix E at 4 m/s for 60 s using a FastPrep-24 5G instrument (MP Biomedicals, Santa Ana, CA, USA), followed by centrifugation (1,100 × g, 5 min). The supernatant (200 µL) was incubated with 20 µL lysozyme (100 mg/mL; Sigma-Aldrich) at 37 °C for 60 min with agitation (1000 rpm), followed by incubation at 80 °C for 15 min with agitation (250 rpm). Samples were then treated with 20 µL Proteinase K and 180 µL Solid Tissue Buffer at 55 °C for 30 min (250 rpm).

#### Colonic biopsies and standards

Were bead-beaten in Lysing Matrix E, using a FastPrep-24 5G instrument (MP Biomedicals), for two runs with a 5-minute cooling interval on ice between runs. Each run consisted of two cycles of 4 m/s for 120 s followed by 300 s rest. Following centrifugation (1,100 × g, 5 min), 100 µL supernatant was incubated with 10 µL lysozyme (100 mg/mL; Sigma-Aldrich, St. Louis, MO, USA) at 37 °C for 60 min with agitation (1000 rpm), followed by incubation with 10 µL Proteinase K and 95 µL Solid Tissue Buffer at 55 °C for 90 min (300 rpm). 

### 16S rRNA gene library preparation

Using the Qubit 1X dsDNA High Sensitivity Assay Kit, DNA concentrations were quantified on a Qubit 3.0 fluorometer (Invitrogen, Thermo Fisher Scientific, Waltham, MA, USA), and diluted to ≤ 5 ng/µL in 10 mM Tris-HCl (pH 8.5).

Amplicon libraries were prepared according to the 16S Metagenomic Sequencing Library Preparation protocol (Illumina, San Diego, CA, USA), using primers targeting the V3–V4 region of the 16S rRNA gene (Invitrogen, Thermo Fisher Scientific) and Illumina DNA/RNA UD indexes (Illumina). A positive control (Microbial Community DNA Standard, D6305 (Zymo Research)) and a no-template control (UV-radiated, DNase free H_2_O) were included in addition to the extraction controls.

The Quick-16S Plus NGS Library Prep Kit was used to create amplicon libraries of both the V3–V4 and the V1–V2 regions of the 16S rRNA gene. Libraries were prepared according to the manufacturer’s instructions and PCR was performed on a CFX96 Real-Time system (Bio-Rad, Hercules, CA, USA), with each run including a positive kit control (Microbial Community DNA Standard, D6305 (Zymo Research)) and a no-template control (UV-radiated, DNase free H_2_O) in addition to extraction controls. Amplicons were pooled and purified according to the manufacturer’s instructions. 

### Sequencing

Paired-end sequencing (2 × 300 bp) was performed on an Illumina NextSeq 2000 system using the NextSeq 1000/2000 P1 XLEAP-SBS Reagent Kit (600 cycles) and 40% PhiX control library (Illumina) spike-in. Basecalling and demultiplexing were carried out using the onboard Real-Time Analysis software (RTA V4.12.2), and DRAGEN BCL Convert (v4.2.7) (Illumina). 

### Bioinformatic analysis

Sequencing reads were processed using nf-core/ampliseq (v2.14.0) [17] and TRANA (v0.3.1), with default parameters unless otherwise specified. Briefly, nf-core/ampliseq performs quality control, primer trimming, denoising and taxonomic classification with DADA2 [18] using the SILVA 138.2 prokaryotic SSU database [19]. TRANA, executed with its short-read workflow, includes quality control, primer trimming, and taxonomic classification using EMU (v3.5.1) [13], with its bundled reference database comprising sequences from NCBI 16S RefSeq and rrnDB [20–22].

In both pipelines, primer trimming was performed using Cutadapt [23]. For Illumina V3–V4 libraries, primers were 5’-CCTACGGGNGGCWGCAG-3’ and 5’-GACTACHVGGGTATCTAATCC-3’. For Zymo V3–V4 libraries, primers were 5’-CCTAYGGGDYGCWGCAG-3’ and 5’-GACTACNVGGGTMTCTAATCC-3’. For nf-core/ampliseq, read truncation lengths were set to 250 bp (forward) and 230 bp (reverse).

For Zymo V1–V2 libraries, primers were 5’-CTGCWGCCHCCCGTAGG-3’ and 3’-AGRGTTYGATYMTGGCTCAG-5’. For nf-core/ampliseq, read truncation lengths were set to 280 bp (forward) for forward reads and 220 bp (reverse), with the parameter dada_taxonomy_rc enabled to allow reverse-complement matching.

### Data analysis

To harmonise annotations across pipelines, synonymous or ambiguous taxonomic labels were collapsed into unified taxon names; *Escherichia* and *Escherichia-Shigella* were merged as “*Escherichia|Escherichia-Shigella”*. The taxon listed as *Lactobacillus fermentum* in the ZymoBIOMICS product sheet was updated to *Limosilactobacillus fermentum* following Zheng et al. [24]. Taxonomic profiles were normalised to relative abundances. Technical duplicates of faecal samples (V3–V4 region) were averaged, while all other samples were treated as independent replicates. Included controls were inspected to assess potential contamination. All analyses were performed in R (version 4.5.1). 

#### Number of detected genera and species

To account for all recovered taxonomic assignments, the number of detected genera and species was compared across protocols, pipelines, and amplicon regions by calculating counts across a predefined series of relative abundance thresholds. Thresholds ranged from 0 to 1% in 0.05% increments and from 2 to 25% in 1% increments. For each sample, the number of taxa exceeding each threshold at the selected taxonomic rank was counted and averaged across region-protocol-pipeline combinations within each sample type. 

#### Microbial diversity analysis

Microbial diversity analysis was performed using the phyloseq (v1.46.0) [25] and vegan (v2.6-8) [26] packages. Alpha diversity was calculated based on the highest available taxonomic resolution for TRANA and at ASV-level for nf-core/ampliseq, reflecting the native output structure of each pipeline. Alpha diversity was evaluated using the Shannon and Simpson indices, with differences assessed using paired Wilcoxon signed-rank tests. For multiple pairwise comparisons, p-values were adjusted using the Benjamini-Hochberg false discovery rate (BH/FDR) method. Beta diversity was evaluated using Bray–Curtis dissimilarity, and ordination was performed using principal coordinate analysis (PCoA). Differences in community composition were assessed using restricted PERMANOVA (999 permutations), with permutations constrained within matched sample pairs. 

#### Evaluation of taxonomic resolution and compositional agreement

Microbial standards (D6300, D6331) were used to assess detection sensitivity, taxonomic resolution, and compositional agreement. Expected relative abundances were obtained from the manufacturer’s reference composition. Observed relative abundances were aggregated at the genus and species levels and compared with the expected composition. Method-dependent deviation was quantified by calculating Bray–Curtis dissimilarities between observed profiles and the corresponding expected composition.

To examine species-level taxonomic resolution and possible misclassification, observed species were identified as expected (present in the manufacturer’s reference composition) or correct genus-level assignments with incorrect species-level classification. Assignments contributing < 1% relative abundance were collapsed to distinguish consistent species-level misclassification from low-level background noise. Species-level abundances were averaged across replicates for each region-protocol-pipeline combination. 

#### Evaluation of methodological consistency in clinical samples

Bray–Curtis dissimilarities were calculated to assess protocol, pipeline, and region effects. Across-region consistency was evaluated by comparing V1–V2 and V3–V4 profiles generated using the same protocol-pipeline combination, while across-protocol consistency was assessed by comparing Illumina- and Zymo-prepared libraries within the same amplicon region. To contextualise method-dependent variation, intra- and inter-individual Bray–Curtis dissimilarities were also calculated.

## Results

### The number of detected genera and species varies across methodological choices

**Fig. 2:**
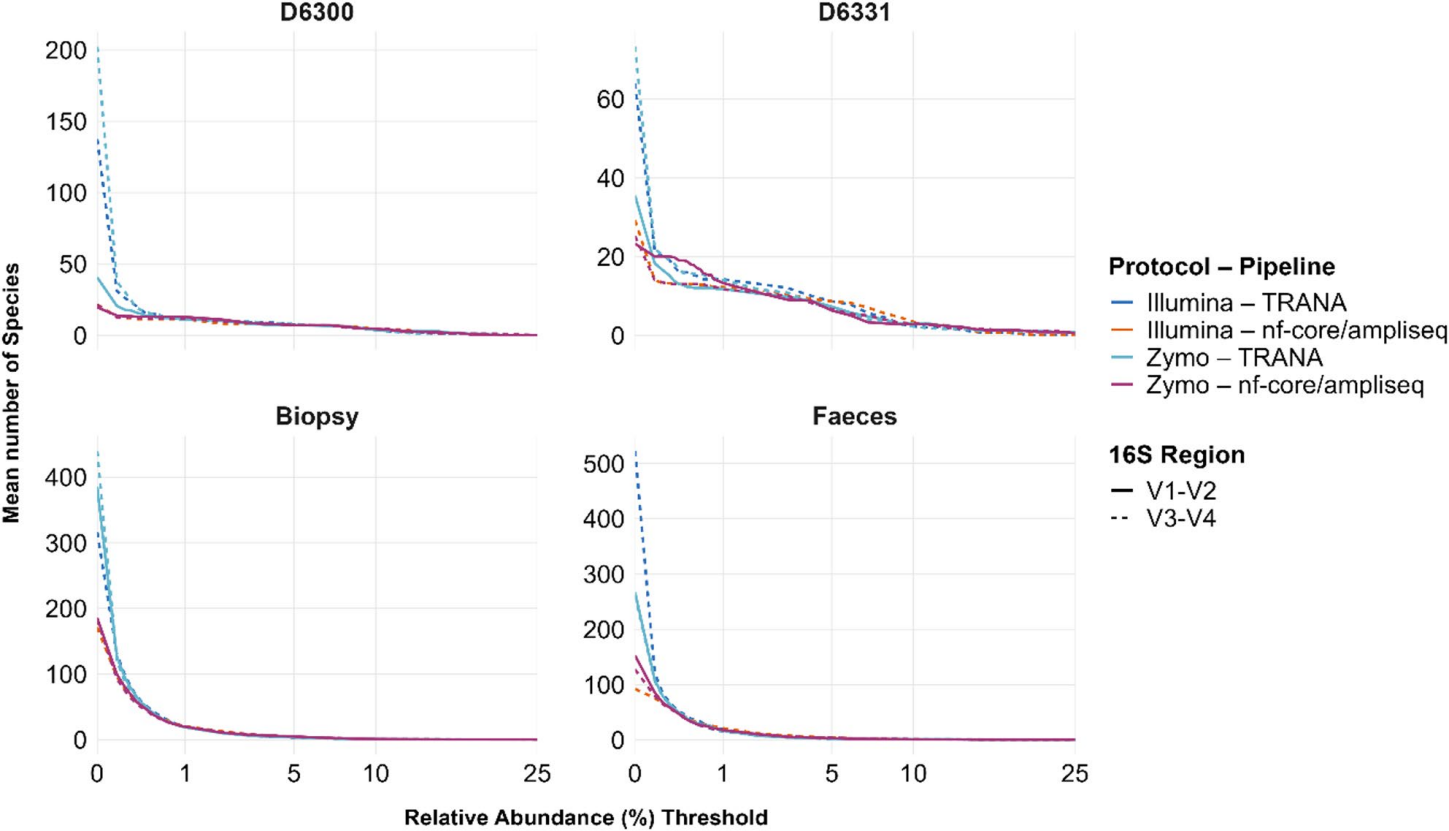
Species detection curves across protocols, pipelines and regions. Detection curves showing the mean number of species recovered across relative abundance thresholds for each sample type, library preparation protocol, bioinformatic pipeline, and 16S amplicon region

The number of detected genera and species taxonomic assignments varied by protocol, pipeline, and amplicon region, with pipeline having the strongest effect (Fig. 2, Supplementary Fig. 1). Counts included all recovered taxonomic assignment at the specified rank and were, for the microbial standards, not restricted to expected taxa. Differences between method combinations decreased at higher relative abundance thresholds. TRANA generally detected more taxonomic assignments than nf-core/ampliseq, especially at low (≤ 1%) relative abundance. Across regions, V3–V4 recovered more taxonomic assignments than V1–V2; however, in faecal samples prepared with the Zymo protocol this effect was minimal. Protocol differences were modest and sample-type dependent.

### Species-level resolution is selectively influenced by bioinformatic pipeline and 16S amplicon region

Relative abundances in microbial standards (D6300 and D6331) were compared with the manufacturer-specified reference composition (Supplementary Table 1).

At the genus level, relative abundances were broadly consistent across both standards, with most samples clustering near expected abundances and comparable resolution across method combinations (Supplementary Fig. 2, Supplementary 1).

**Fig. 3:**
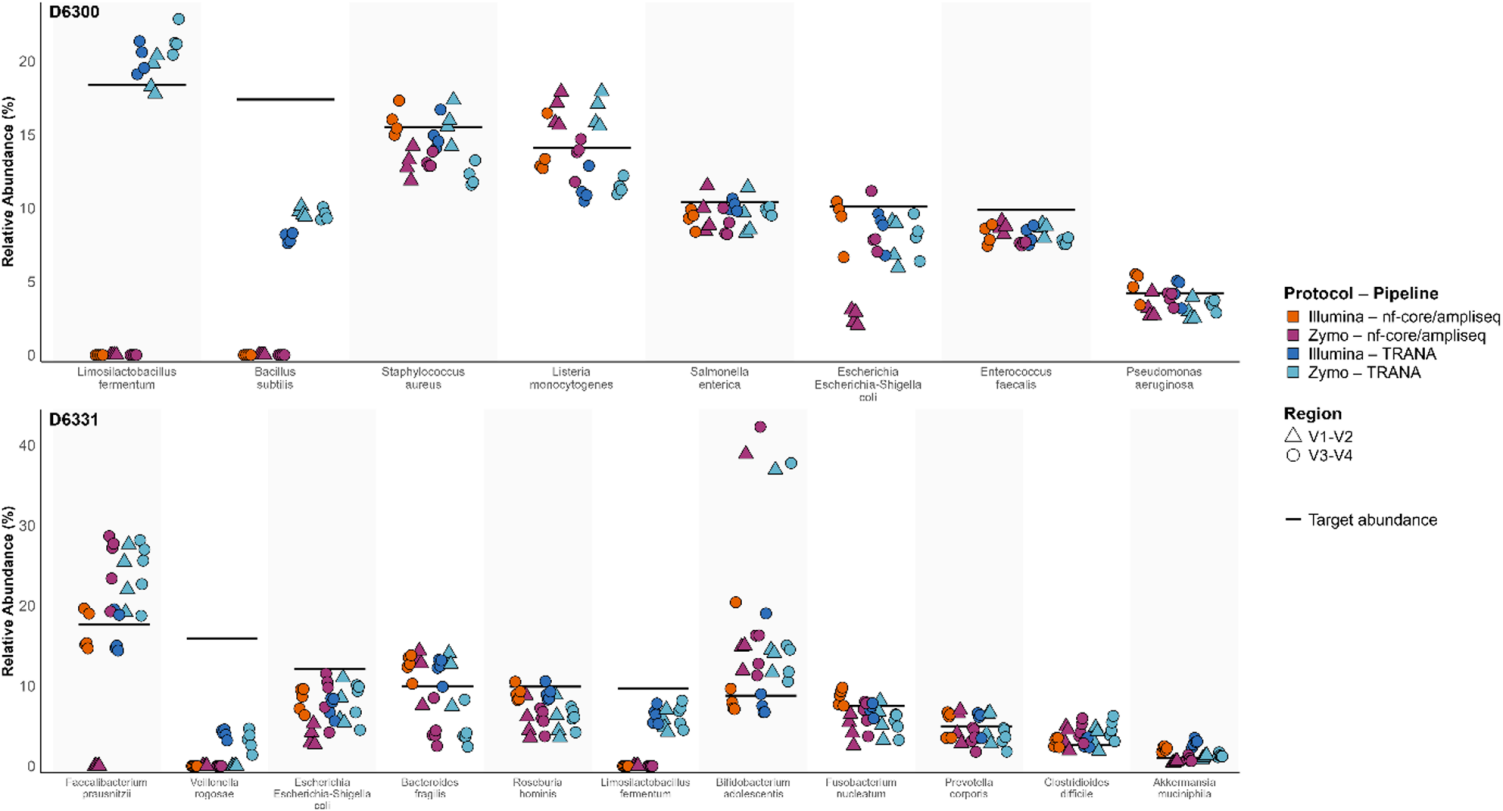
Relative abundance of expected species in microbial standards. Relative abundance of expected species in microbial standards D6300 (top) and D6331 (bottom) across library preparation protocols, bioinformatic pipelines, and 16S amplicon regions. Only species with expected abundance ≥ 0.1% are shown. Horizontal lines indicate expected target abundances as specified by the manufacturer

Species-level resolution varied markedly across method combinations (Fig. 3, Supplementary 1). nf-core/ampliseq failed to resolve *Limosilactobacillus fermentum*, *Bacillus subtilis*, and *Veillonella rogosae* across all method combinations; failed to resolve *Faecalibacterium prausnitzii* in the V1–V2 region; and underestimated *Escherichia|Escherichia-Shigella coli* in the V1–V2 region. TRANA recovered *L. fermentum* across all method combinations at near-expected abundances, underestimated *B. subtilis*, with slightly greater deviation for the Illumina protocol, and resolved *V. rogosae* only in the V3–V4 region, where it was recovered at lower-than-expected abundance. For *F. prausnitzii*, greater replicate variability was observed with the Zymo protocol across pipelines. For *Bifidobacterium adolescentis*, two Zymo-derived samples exhibited notably higher abundances than expected at both the genus and species level for both pipelines.

Taxa present at low abundances (< 0.1%) displayed similar resolution at genus and species level (Supplementary Fig. 3, Supplementary 1). Detection was generally absent for the V1–V2 region and sporadic for the V3–V4 region for several low-abundance taxa, with variable recovery across protocols.

**Fig. 4:**
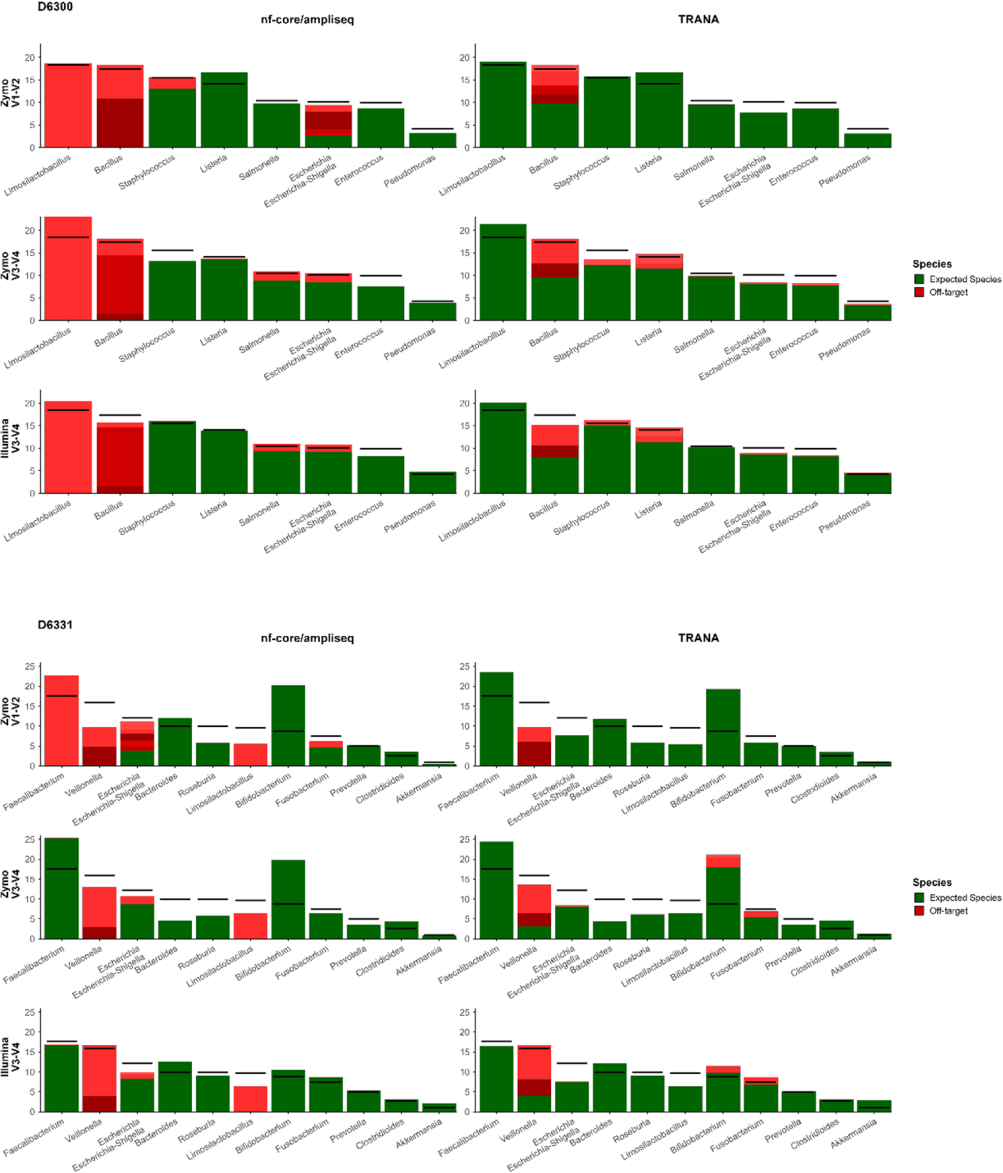
Species-level composition within expected genera in microbial standards. Mean relative abundances of expected genera and their constituent species in the microbial standards D6300 (top) and D6331 (bottom) across library preparation protocols, bioinformatic pipelines, and 16S amplicon regions. Low-abundance (< 1%) incorrect species-level assignments within the expected genus were collapsed prior to calculating mean relative abundances. Stacked bars display correctly assigned species (green) and incorrect species-level assignments within the same expected genus (red tones). Different red tones are used to visually separate incorrect species-level assignments within each genus (and do not represent the same species across genera or panels). Black horizontal lines indicate the expected target abundances as specified by the manufacturer

To further characterise species-level performance, assignments were examined to identify species-level misclassifications within expected genera (Fig. 4, Supplementary 2). nf-core/ampliseq failed to resolve species within several expected genera: *Limosilactobacillus* was retained at the genus level; *Bacillus* was retained at the genus level, particularly for Zymo-prepared samples, or reassigned to alternative species within the genus; and *Veillonella* was similarly misassigned, being predominantly classified as *V. parvula* or retained at the genus level. *Staphylococcus* was more frequently retained at the genus level in the V1–V2 region, whereas the V3–V4 region predominantly recovered the expected species. *Escherichia|Escherichia-Shigella coli* was substantially underestimated in the V1–V2 region but recovered closer to expected abundances in the V3–V4 region, with misassigned species exceeding 1% abundance differing between the microbial standards. For *Salmonella*, the V3–V4 region yielded slightly fewer genus-level assignments than V1–V2 in D6300. *Fusobacterium* showed partial genus-level retention with the V1–V2 region but predominantly correct species-level assignment with the V3–V4 region.

TRANA correctly classified *B. subtilis* across all protocol-region combinations, though underestimating its abundance. Misclassification patterns differed between regions, with the V1–V2 region showing broader redistribution across multiple alternative *Bacillus* species. *B. subtilis* remained the dominant species-level assignment across regions. *Veillonella rogosae* was not resolved with the V1–V2 region, while the V3–V4 region resolved *V. rogosae* at lower-than-expected abundances with residual redistribution within the genus. For *Staphylococcus aureus*, *Listeria monocytogenes* and *B. adolescentis*, the V1–V2 region predominantly recovered the expected species, whereas the V3–V4 region classified additional low-abundance species. A similar region-dependent pattern was observed for *Fusobacterium nucleatum*, where correct species-level assignment predominated, with additional species detected primarily with the V3–V4 region. 

### Bioinformatic pipeline significantly affects alpha diversity in biopsies

**Fig. 5:**
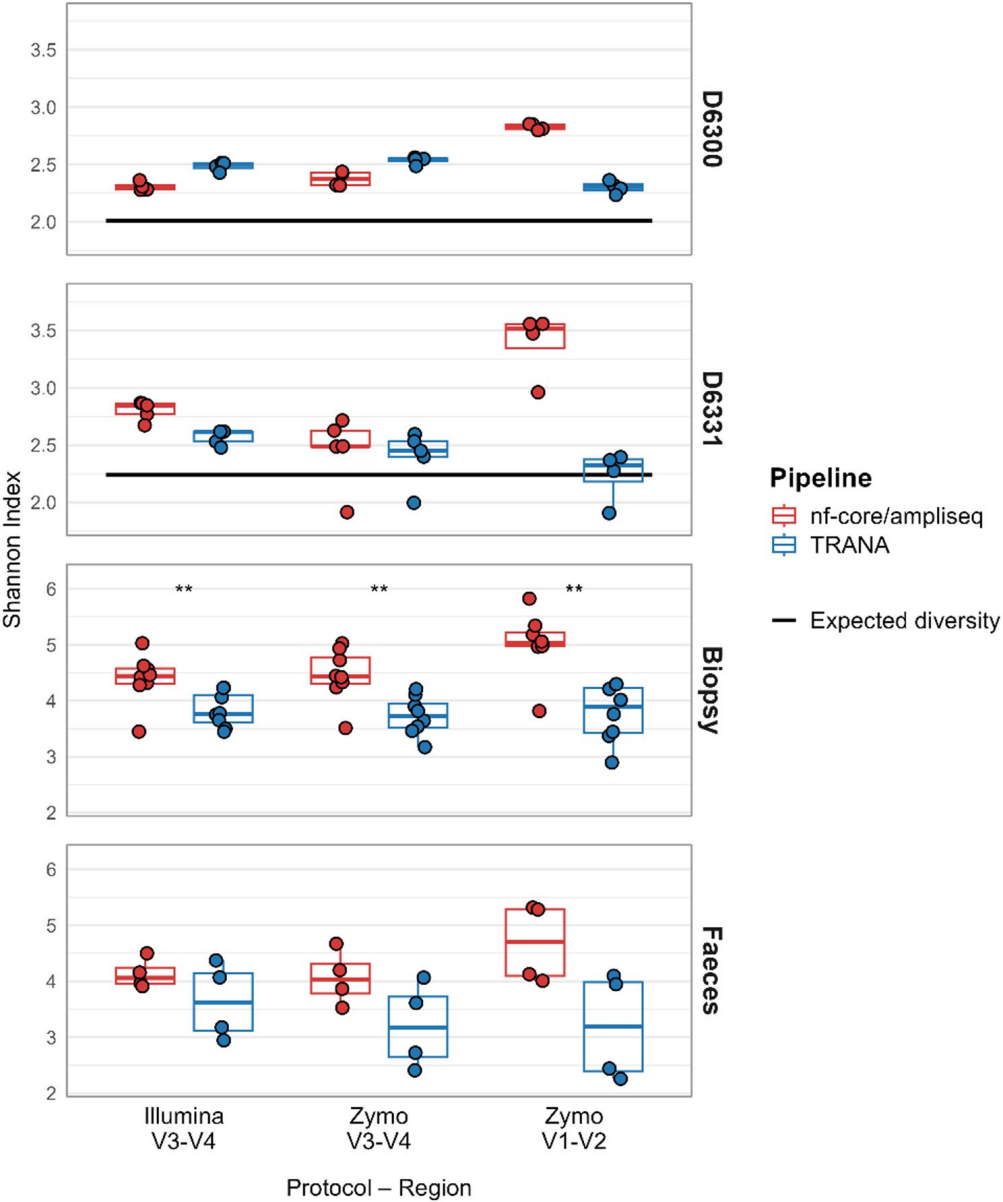
Shannon diversity across protocols, pipelines, regions and sample types. Shannon diversity index across sample types, library preparation protocols, bioinformatic pipelines, and 16S amplicon regions. Horizontal black lines indicate the expected Shannon diversity of the microbial standards (D6300 and D6331), calculated from the manufacturer-specified composition. Paired Wilcoxon test results are shown between pipelines within each protocol-region combination and are annotated with significance markers (* *p* < 0.05, ** *p* < 0.01, *** *p* < 0.001)

Alpha diversity was evaluated using the Shannon and Simpson indices for each sample type (Fig. 5, Supplementary Fig. 4, Supplementary 3–4).

For D6300, Shannon indices clustered around ~ 2.30–2.83. The V3–V4 region showed limited between-pipeline differences, with slightly higher values for TRANA than nf-core/ampliseq, whereas the V1–V2 region yielded higher Shannon values with nf-core/ampliseq relative to TRANA. Simpson indices displayed the same pattern. For D6331, nf-core/ampliseq produced slightly higher Shannon and Simpson values than TRANA across protocols, with the largest separation again observed for the V1–V2 region. Comparison with diversity calculated from the manufacturer-defined composition showed overall close agreement, but the V1–V2 region exhibited greater method dependence for both indices.

For biopsy and faecal samples, nf-core/ampliseq generally returned higher alpha diversity estimates than TRANA for both indices. In biopsy samples, paired Wilcoxon tests comparing pipelines within each protocol-region indicated a significant pipeline effect for both indices (Supplementary 4). Broader pairwise comparisons across all region-protocol-pipeline combinations confirmed a between-pipeline difference for Shannon after BH/FDR correction (adjusted *p* ~ 0.047) (Supplementary 5). No significant differences were detected for faecal samples. 

### Microbial community profiles are predominantly influenced by the choice of bioinformatic pipeline

**Fig. 6:**
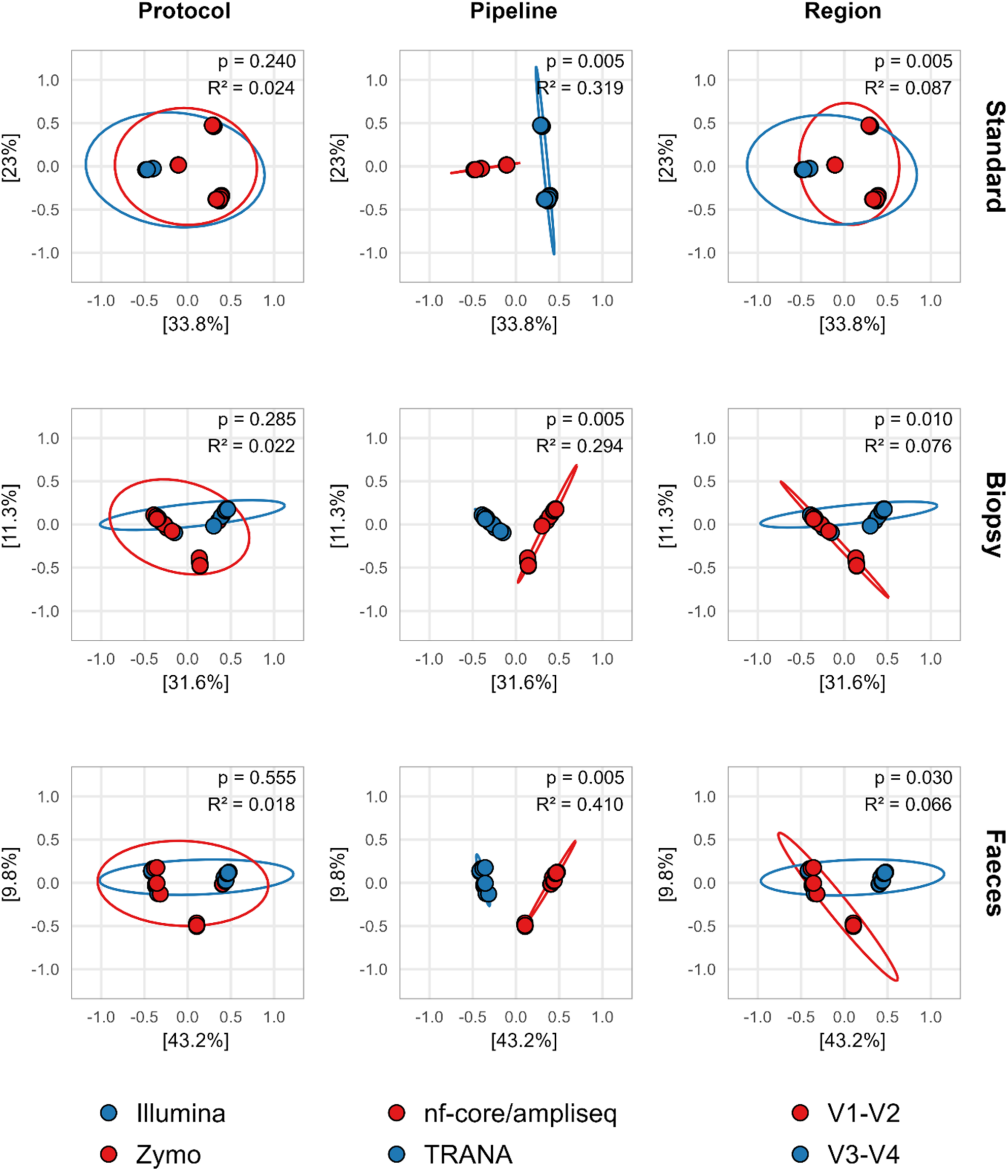
Principal coordinate analysis (PCoA) of Bray–Curtis dissimilarities across protocols, pipelines, regions and sample types. Principal coordinate analysis of Bray–Curtis dissimilarities. Ellipses denote 95% confidence interval. Paired PERMANOVA p-values and R^2^ statistics indicate the proportion of variance explained by each factor within each sample type

Beta diversity was assessed using Principal coordinate analysis (PCoA) of Bray–Curtis dissimilarities. Pipeline choice had the strongest influence on microbial community composition, across all sample types (microbial standards, biopsies and faeces) (Fig. 6).

Samples processed with TRANA and nf-core/ampliseq formed clearly separated clusters, with a statistically significant effect of pipeline choice (PERMANOVA: *p* = 0.005). Pipeline consistently accounted for the largest proportion of variance (R^2^ = 0.294–0.319).

The effect of amplicon region was intermediate but consistent. All sample types exhibited significant region-dependent shifts in ordination space (PERMANOVA: *p* < 0.05), although the R^2^ values were small (Microbial standards: R^2^ = 0.087, Biopsies: R^2^ = 0.076, Faeces: R^2^ = 0.066).

Protocol showed only a minor effect on community composition. No significant separation was observed in any sample type (PERMANOVA: *p* = 0.240–0.555), and R^2^ values were low (R^2^ = 0.018–0.024), indicating minimal difference between protocols. 

### Bioinformatic pipeline influences species-level compositional estimates

To evaluate how closely each method combination recovered the expected composition of the microbial standards, Bray–Curtis dissimilarity was calculated between each processed sample and the manufacturer-specified composition (Supplementary Table 1).

At the genus level, Bray–Curtis dissimilarities were uniformly low for D6300, whereas D6331 displayed slightly higher distances, particularly for one sample prepared with the Zymo protocol for both regions (Supplementary Fig. 5).

**Fig. 7:**
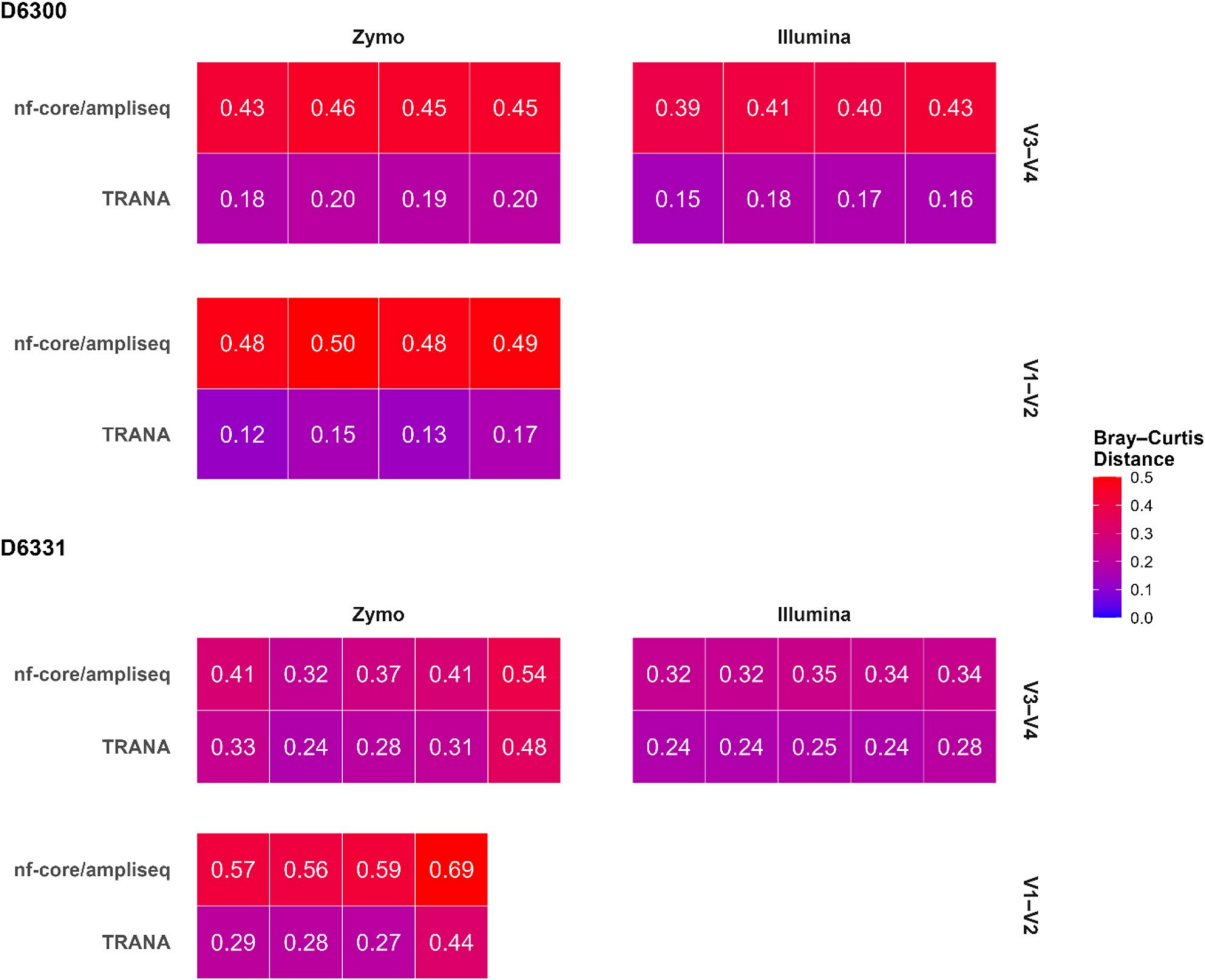
Bray–Curtis dissimilarities to expected composition at species-level. Each tile represents the Bray–Curtis dissimilarity for a single processed sample relative to the manufacturer-specified composition. Distances are shown across all combinations of library preparation protocol, bioinformatic pipeline, and 16S amplicon region. Lower values indicate closer agreement with the expected compositions

At the species level, systematic differences were observed (Fig. 7). Across both microbial standards (D6300 and D6331), nf-core/ampliseq consistently produced higher Bray–Curtis dissimilarities than TRANA for all protocol-region combinations, indicating poorer agreement with the expected composition. TRANA yielded substantially lower dissimilarities, typically less than half of those observed with nf-core/ampliseq. For D6300, nf-core/ampliseq yielded distances of 0.39–0.50, compared with 0.12–0.20 for TRANA. For D6331, nf-core/ampliseq distances ranged from 0.32 to 0.69, whereas TRANA remained lower (0.24–0.44). Replicates within each protocol-region-pipeline combination were consistent, indicating that these differences were systematic rather than driven by replicate-level variability.

Amplicon region effects were modest, with directionality dependent on pipeline choice. TRANA generally produced lower dissimilarities for the V1–V2 region than for the V3–V4 region, although the differences were small. nf-core/ampliseq consistently produced higher dissimilarities for the V1–V2 region than the V3–V4 region.

Differences in Bray–Curtis dissimilarity between protocols were broadly similar, with no systematic advantage observed for either protocol. This pattern was consistent at both genus and species level, although one sample prepared with the Zymo protocol for both regions displayed elevated dissimilarity compared with the general genus-level trend. (Supplementary Fig. 5, Fig. 7) 

### Microbial community profiles in biopsy and faecal samples show greater inter-individual than method-associated variation

To contextualise methodological variation, Bray–Curtis dissimilarities across protocol, amplicon region and pipeline were compared with inter-individual dissimilarities.

**Fig. 8:**
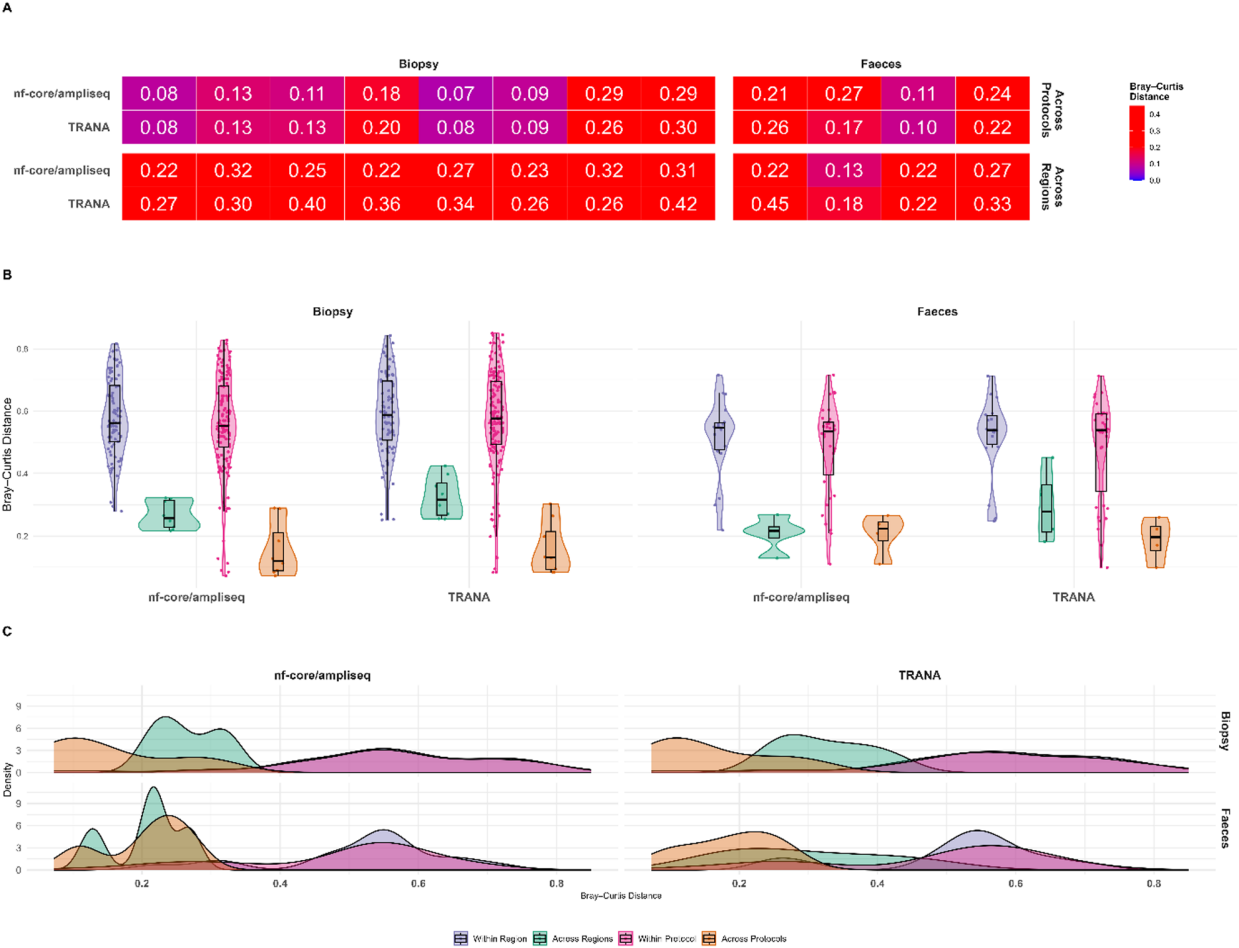
Bray–Curtis dissimilarities across protocols, pipelines and regions in clinical samples. For faecal samples, which contain technical replicates, abundances were averaged prior to distance calculation. **A** Pairwise Bray–Curtis dissimilarities across protocols and across regions for each pipeline. **B** Distribution of intra-sample and inter-sample Bray–Curtis dissimilarities across pipelines and sample types, visualised as violin plots with overlaid boxplots. **C** Density plots showing the overall distribution of dissimilarities within each pipeline and sample type, encompassing both across- and within-protocol and region comparisons. Across all panels, lower values indicate closer agreement in recovered community composition

Across clinical sample types, Bray–Curtis dissimilarities were generally low when comparing the two protocols, although faecal samples showed slightly higher values than biopsies (Fig. 8). Comparisons across amplicon regions yielded higher dissimilarities; the magnitude of the region effect varied by sample type and pipeline, with TRANA generally demonstrating slightly higher region-associated distances than nf-core/ampliseq. Biological variation between samples was substantially greater than methodological variation, as inter-sample Bray–Curtis distances were markedly higher than within-region or within-protocol distances.

## Discussion

In this study, we systematically evaluated how library preparation protocol, 16S amplicon region, and bioinformatic pipeline influence taxonomic resolution, detection sensitivity, and relative abundance estimates in short-read 16S rRNA sequencing. Using microbial standards, together with clinically relevant samples, we demonstrate that, within the workflows evaluated here, pipeline choice was the dominant driver of methodological variation, particularly at the species level. Amplicon region had a secondary effect, whereas protocol effects were comparatively small.

Beta diversity analyses based on Bray–Curtis dissimilarities showed that pipeline choice accounted for the largest proportion of variation in microbial composition across all sample types. Amplicon region had a secondary but consistent effect, reflecting modest but significant region-dependent differences. Protocol differences were small, indicating broad comparability in overall community composition. These findings indicate that downstream analytical decisions contribute substantially to variation in inferred community composition, in some cases exceeding upstream experimental effects.

While beta diversity captures differences in overall community structure, it does not directly assess whether reconstructed profiles match the known reference composition. Using defined microbial standards, Bray–Curtis dissimilarities to the expected composition indicated generally good agreement at the genus level across all method combinations. At the species level systematic differences were observed. nf-core/ampliseq consistently produced higher dissimilarities than TRANA across all protocol-region combinations, indicating poorer agreement with the expected community composition. Amplicon region effects on species-level agreement with the expected composition were modest and pipeline-dependent. While TRANA generally yielded slightly lower dissimilarities for the V1–V2 region than for V3–V4, the opposite trend was observed for nf-core/ampliseq, indicating that region-specific effects on species-level agreement also depend on pipeline. Protocol effects were comparatively small and inconsistent, with no systematic advantage observed for either protocol. The elevated Bray–Curtis dissimilarity observed for two replicate samples was attributable to overrepresentation of *B. adolescentis*. Similar deviations were not observed in other replicates, indicating replicate-specific variability rather than a systematic protocol effect.

For the dominant taxa in the microbial standards, taxonomic resolution differed between genus and species level. At the genus level, all method combinations recovered the expected genera and produced broadly comparable relative abundance estimates, indicating that genus-level taxonomic assignment is largely insensitive to protocol, amplicon region, and pipeline choice. At species level, however, taxonomic resolution was strongly influenced by pipeline, with TRANA more consistently recovering expected species than nf-core/ampliseq. For nf-core/ampliseq, a systematic genus-level retention was observed, as several taxa were retained at the genus level or redistributed across congeneric species, thereby limiting species-level interpretability despite correct higher-level classification. Low-abundance taxa showed broadly consistent profiles at genus and species level, but recovery varied across protocols and regions. This pattern is consistent with variable detection of low-abundance taxa in 16S amplicon sequencing, where recovery becomes increasingly stochastic near the limit of detection and more susceptible to small methodological effects, including region-dependent primer bias and library preparation-associated variability [7].

In clinically derived samples, biological variation between individuals substantially exceeded the technical variation associated with protocol, amplicon region, or pipelines. Across both biopsies and faecal samples, comparison between protocols yielded generally low Bray–Curtis dissimilarities, indicating that protocol choice contributed relatively little to shifts in overall community composition. Between regions, Bray–Curtis dissimilarities were moderately higher, implying that choice of amplicon region contributes a more substantial technical source of variation at the community level. The magnitude of this region-associated effect varied by sample type and pipeline, with TRANA generally demonstrating higher region-associated dissimilarities than nf-core/ampliseq. Nonetheless, these technical effects remained smaller than the dissimilarities observed between individual biological samples.

The number of detected genera and species was strongly influenced by pipeline, with TRANA detecting more low-abundance taxa than nf-core/ampliseq, while effects of the amplicon region were secondary. The higher number of detected genera and species obtained with TRANA likely reflects increased sensitivity to low abundance features rather than true biological differences, indicating that pipeline choice exerts a strong influence on apparent richness. This pattern suggests that TRANA has a more permissive retention of rare features, some of which may represent ambiguously assigned taxa at very low abundances. This behaviour is consistent with the probabilistic nature of EMU, which can result in broader distribution of low-abundance reads across multiple taxa [13]. Importantly, differences in genus- and species-level assignments counts should be interpreted with caution, as a higher number of detected genera or species does not necessarily correspond to finer taxonomic discrimination, nor does a lower number indicate loss of biologically relevant information. Rather, these differences may reflect distinct trade-offs in how sequences are handled near the limit of detection [27, 28]. Although controls were inspected, no formal control-based decontamination step was applied; therefore, low-abundance assignments may also be affected by background contamination or analytical noise. While taxon count provides a measure of richness, alpha diversity metrics such as the Shannon [29] and Simpson [30] indices additionally incorporate evenness by weighting taxa according to their relative abundances. As a result, these indices are less influenced by the presence or absence of rare features. Consistent with this, the higher richness observed with TRANA did not translate into uniformly higher alpha diversity estimates. Instead, nf-core/ampliseq generally yielded higher Shannon and Simpson values, particularly in biopsy samples.

The discrepancies in taxonomic detection and resolution observed between the pipelines likely reflect differences in taxonomic assignment strategy and reference database composition. nf-core/ampliseq, when used with its default DADA2-based workflow, performs model-based inference of exact amplicon sequence variants (ASVs) prior to taxonomic classification. Subsequent annotation is constrained by sequence similarity and the discriminatory capacity of the targeted 16S rRNA region, which may limit reliable species-level resolution [12, 18]. In contrast, TRANA employs EMU, which applies an expectation-maximisation framework to estimate taxonomic abundances by modelling the likelihood of read origin across multiple candidate reference sequences. This probabilistic approach iteratively redistributes ambiguous alignments and refines abundance estimates [13]. These distinct approaches likely underlie the higher species-level Bray–Curtis dissimilarities and genus-level retention observed for nf-core/ampliseq, as well as the increased detection of low-abundance taxa and apparent richness seen with TRANA.

This study has several limitations. First, the number of clinical samples was limited, and although consistent patterns were observed across sample types, larger cohorts would be required to fully characterise method-dependent variability across diverse microbiome states. While defined microbial standards provide a valuable benchmark for assessing taxonomic assignment and agreement with expected composition, they cannot fully capture the compositional complexity or abundance distributions encountered in clinical material. In addition, DNA extraction bias was not independently evaluated and may have contributed to some observed discrepancies in microbial composition. As the same extraction workflow was applied across all samples, extraction-associated bias was controlled across method comparisons but was not separated from downstream workflow effects. The pipelines were run largely with default parameters to reflect typical usage, and alternative parametrisation may yield different results.

## Conclusions

Our findings demonstrate that bioinformatic pipeline choice is a major determinant of inferred taxonomic resolution and compositional agreement in short-read 16S rRNA gene sequencing. These results underscore the importance of careful, study-specific workflow selection and caution in cross-study comparisons using different amplicon regions, library preparation protocols, or bioinformatic processing. Within this study, TRANA showed more consistent species-level agreement with expected microbial standard compositions than nf-core/ampliseq. Given the minor differences observed between protocols, selection may be guided by logistical considerations, with the Zymo protocol offering practical advantages in reduced hands-on time and flexibility in region selection.

## Supplementary Information

Supplementary Material 1: Additional file 1. Relative abundance values for all genera and species in microbial standards D6300 and D6331 across library preparation protocols, bioinformatic pipelines, and 16S amplicon region, including individual replicate values and mean for every protocol-pipeline-region combination.

Supplementary Material 2: Additional file 2. Relative abundances of expected species and incorrectly assigned species within the expected genus for microbial standards D6300 and D6331 across library preparation protocols, bioinformatic pipelines, and 16S amplicon regions. For each genus, expected species and off-target species are reported separately, with off-target species contributing <1% collapsed prior to calculating mean relative abundances.

Supplementary Material 3: Additional file 3. Alpha diversity metrics (Shannon and Simpson indices) across sample types, library preparation protocols, bioinformatic pipelines, and 16S amplicon regions, including expected diversity of the microbial standards calculated from the manufacturer-specified composition.

Supplementary Material 4: Additional file 4. Paired Wilcoxon signed-rank test for alpha diversity metrics (Shannon and Simpson) comparing TRANA and nf-core/ampliseq within each Protocol-Region group across all sample types.

Supplementary Material 5: Additional file 5. Supplementary 5. Pairwise Wilcoxon signed-rank test for alpha diversity metrics (Shannon and Simpson) across all Pipeline-Protocol-region combinations and sample types, including raw and BH-FDR adjusted p-values.

Supplementary Material 6.

## Data Availability

The sets supporting the conclusions of this article are available in the NCBI Sequence Read Archive (SRA) repository under accession number PRJNA1439785, with BioSample accessions SAMN56592478-SAMN5659254.
