# Supplementary material for: Comparison of library preparation protocols and bioinformatic pipelines in high-throughput 16S rRNA gene sequencing
Source: BMC Microbiol. 2026 Jul 1;26:580. doi: 10.1186/s12866-026-05344-6 (PMC13326092; doi:10.1186/s12866-026-05344-6)
Supplement: Supplementary file 6 — Supplementary Material 6. [file 12866_2026_5344_MOESM6_ESM.docx]

# Supplementary Material

**Supplementary Table 1. Theoretical species composition of microbial standards D6300 and D6331**
Theoretical relative abundances of microbial species in the ZymoBIOMICS® Microbial Community Standard (D6300) and ZymoBIOMICS® Gut Microbiome Standard (D6331) (16S-only; Zymo Research).

| D6300 | |  | D6331 | | |
| --- | --- | --- | --- | --- | --- |
| Species | Theoretical Composition (%) |  | Species | Theoretical Composition (%) | |
| *Pseudomonas aeruginosa* | 4.2 |  | *Faecalibacterium prausnitzii* | 17.63 | |
| *Escherichia  coli* | 10.1 |  | *Veillonella  rogosae* | 15.87 | |
| *Salmonella  enterica* | 10.4 |  | *Roseburia  hominis* | 9.89 | |
| *Limosilactobacillus fermentum** | 18.4 |  | *Bacteroides  fragilis* | 9.94 | |
| *Enterococcus faecalis* | 9.9 |  | *Prevotella  corporis* | 4.98 | |
| *Staphylococcus aureus* | 15.5 |  | *Bifidobacterium adolescentis* | 8.78 | |
| *Listeria monocytogenes* | 14.1 |  | *Fusobacterium nucleatum* | 7.49 | |
| *Bacillus  subtilis* | 17.4 |  | *Limosilactobacillus fermentum** | 9.63 | |
| *Saccharomyces cerevisiae* | N/A |  | *Clostridioides difficile* | 2.62 | |
| *Cryptococcus neoformans* | N/A |  | *Akkermansia muciniphila* | 0.97 | |
|  | |  | *Methanobrevibacter smithii* | 0.066 | |
|  | |  | *Salmonella  enterica* | 0.009 | |
|  | |  | *Enterococcus faecalis* | 0.0009 | |
|  | |  | *Clostridium perfringens* | 0.0002 | |
|  | |  | *Escherichia  coli* | 12.12 | |
|  | |  | *Candida  albicans* | N/A | |
|  | |  | *Saccharomyces cerevisiae* | N/A | |
| * Listed as Lactobacillus fermentum in ZymoBIOMICS® product sheet; updated following Zheng et al (24). | | | | |  |


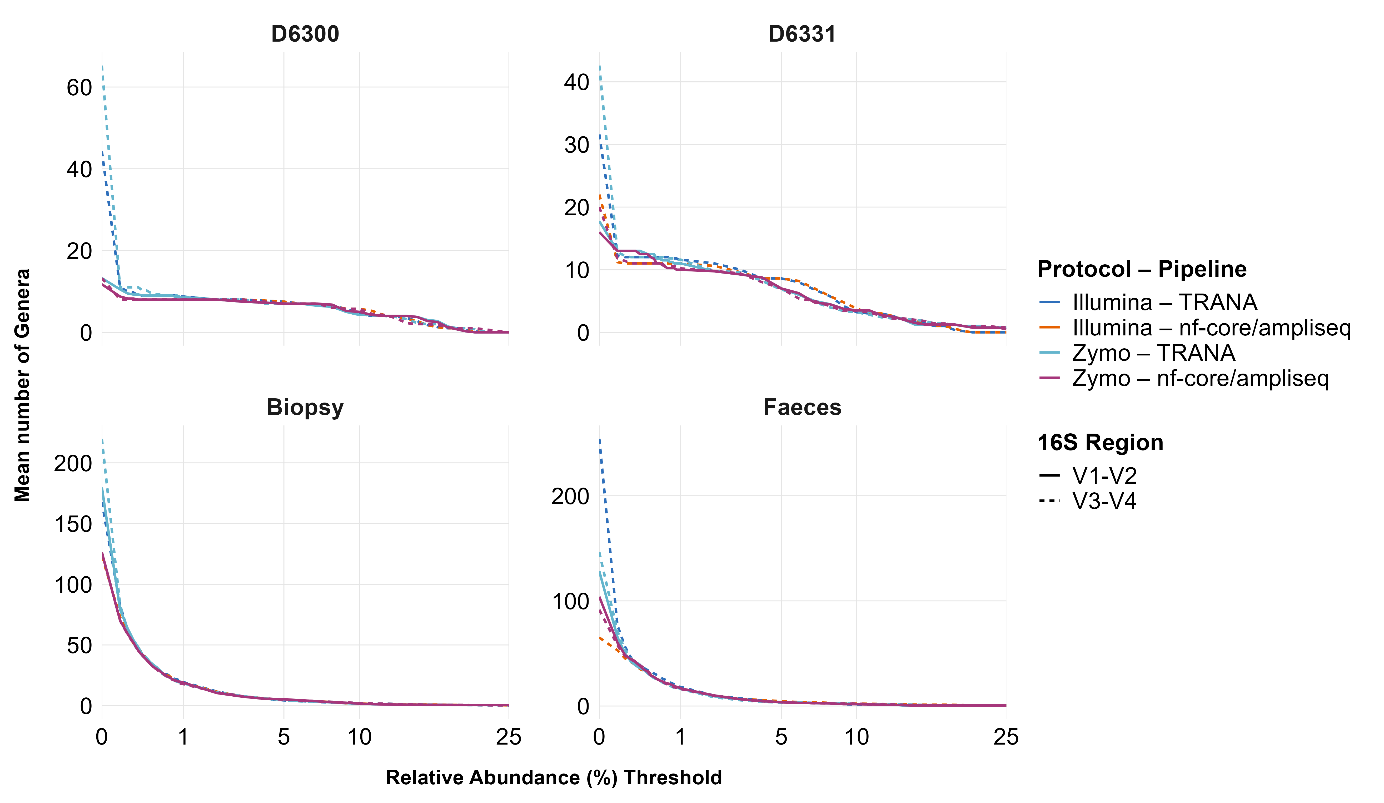


**Supplementary Fig. 1.** **Genus detection curves across protocols, pipelines and regions**Detection curves showing the mean number of genera recovered across relative abundance thresholds for each sample type, library preparation protocol, bioinformatic pipeline, and 16S amplicon region.


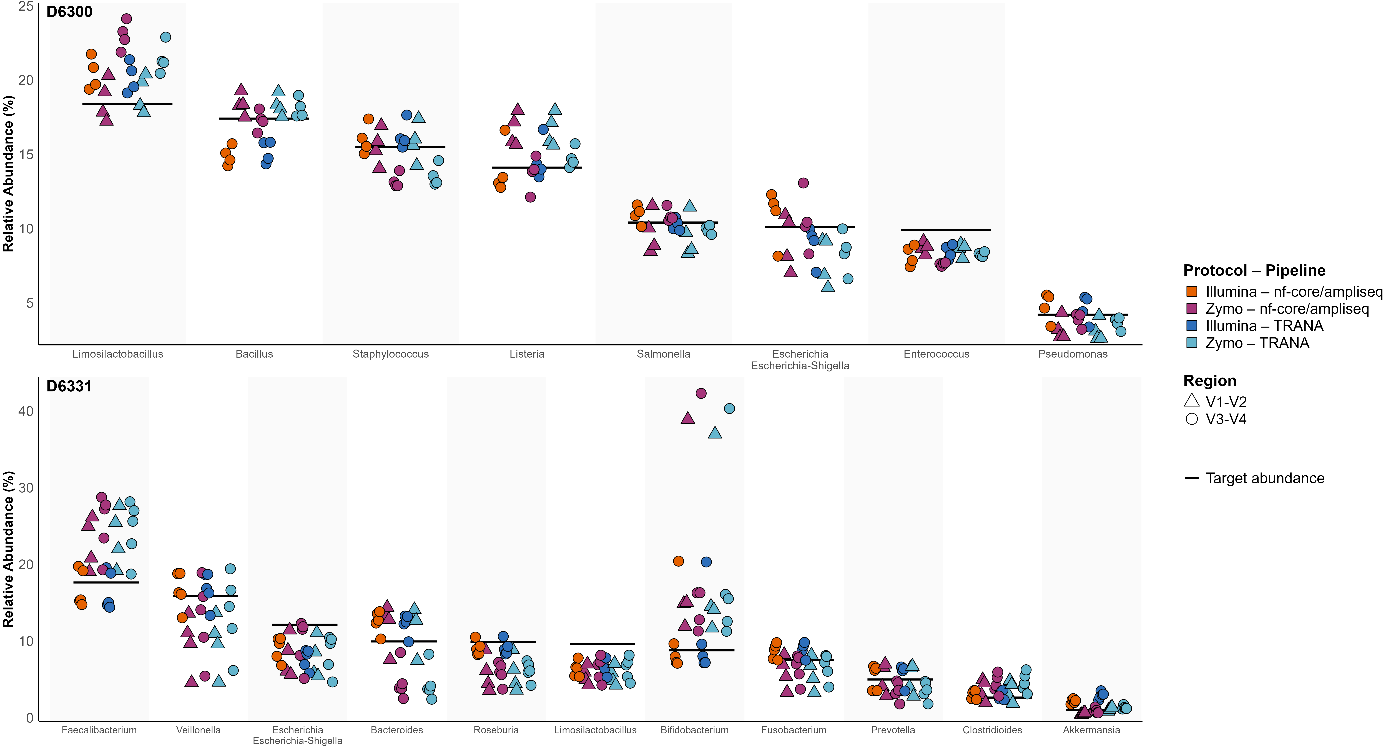


**Supplementary Fig. 2. Relative abundance of expected genera in microbial standards**
Relative abundance of expected genera in microbial standards D6300 (top) and D6331 (bottom) across library preparation protocols, bioinformatic pipelines, and 16S amplicon regions. Only genera with expected abundance ≥ 0.1% are shown. Horizontal lines indicate expected target abundances as specified by the manufacturer.


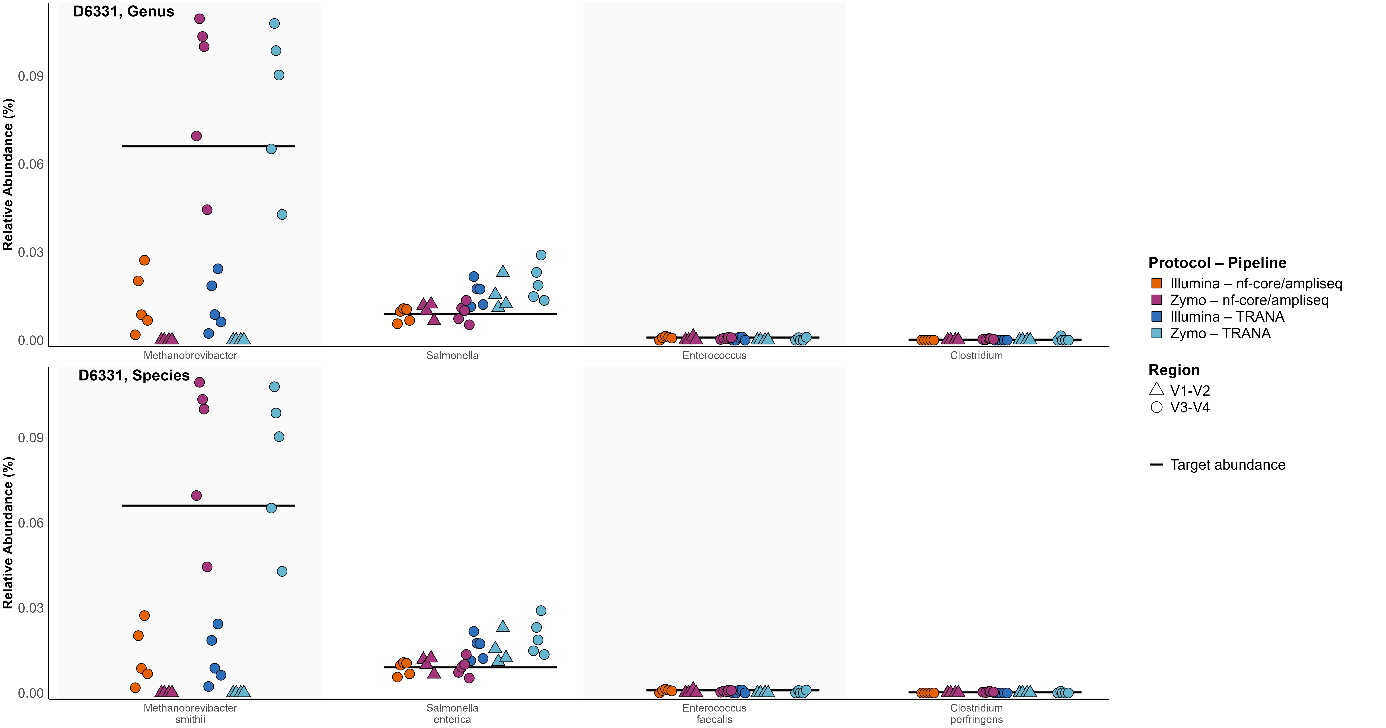


**Supplementary Fig. 3. Relative abundance of expected low-abundance taxa in microbial standard D6331** Relative abundance of expected low-abundance taxa (<0.1%) in microbial standard D6331 across library preparation protocols, bioinformatic pipelines, and 16S amplicon regions. Horizontal lines indicate expected target abundances as specified by the manufacturer.


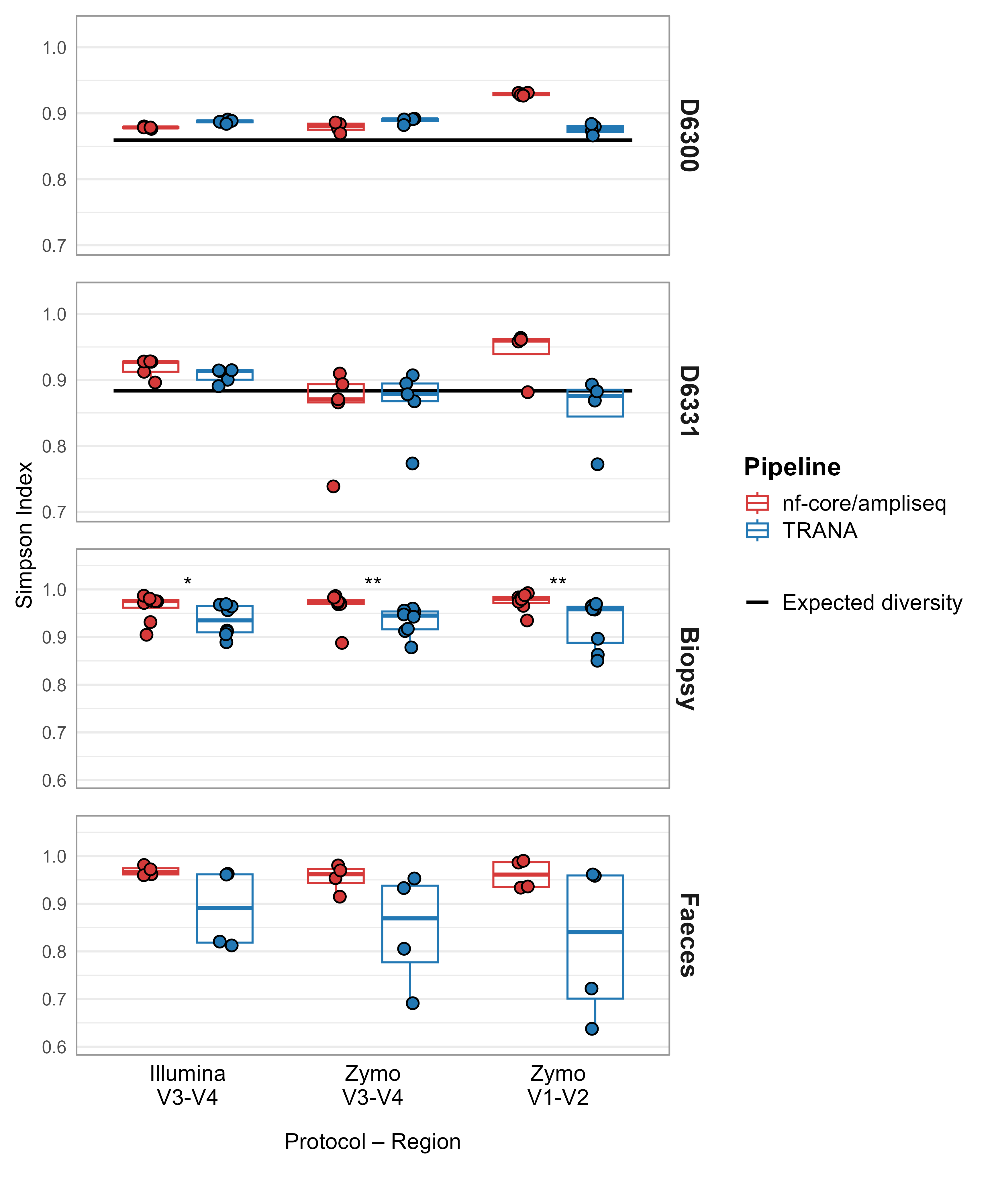


**Supplementary Fig. 4. Simpson diversity across protocols, pipelines, regions and sample types**
Simpson diversity index across sample types, library preparation protocols, bioinformatic pipelines, and 16S amplicon regions. Horizontal black lines indicate the expected Simpson diversity of the microbial standards (D6300 and D6331), calculated from the manufacturer-specified composition. Paired Wilcoxon test results are shown between pipelines within each protocol-region combination and are annotated with significance markers (* p<0.05, ** p<0.01, *** p<0.001).


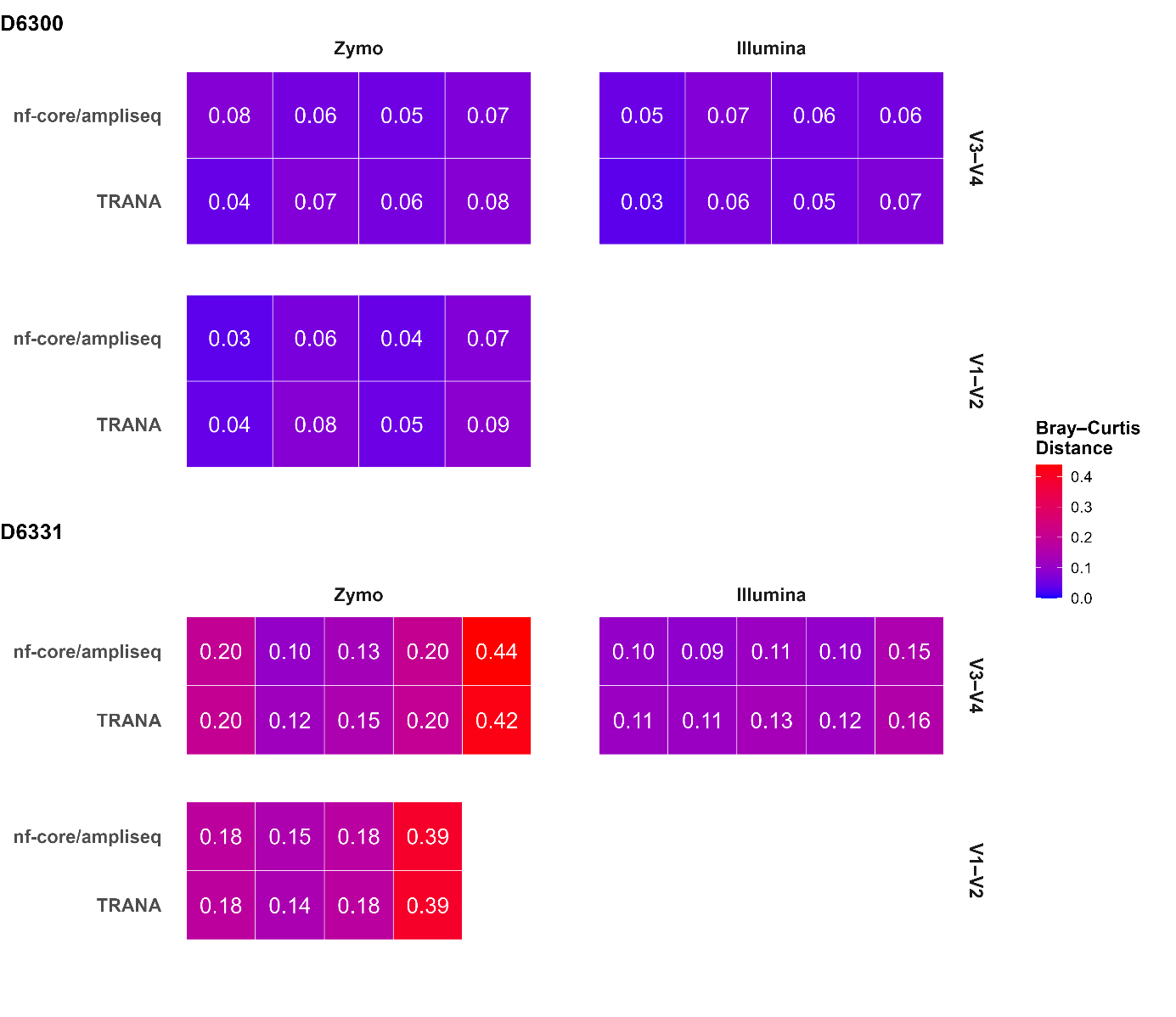


**Supplementary Fig. 5. Bray–Curtis dissimilarities to expected composition at genus-level**
Bray–Curtis dissimilarities to expected composition of microbial standards at genus level. Each tile represents the Bray–Curtis dissimilarity for a single processed sample relative to the manufacturer-specified composition. Distances are shown across all combinations of library preparation protocol, bioinformatic pipeline, and 16S amplicon region. Lower values indicate closer agreement with the expected compositions.
